# Spatiotemporal Variation of Soil Erosion Characteristics in the Qinghai Lake Basin Based on the InVEST Model

**DOI:** 10.3390/ijerph20064728

**Published:** 2023-03-08

**Authors:** Zhen Chen, Xiaohong Gao, Zhifeng Liu, Kelong Chen

**Affiliations:** 1 Key Laboratory of Tibetan Plateau Land Surface Processes and Ecological Conservation (Ministry of Education), Qinghai Normal University, Xining 810008, China; 2 Qinghai Province Key Laboratory of Physical Geography and Environmental Process, Qinghai Normal University, Xining 810008, China; 3 College of Geographical Science, Qinghai Normal University, Xining 810008, China; 4 Center for Human-Environment System Sustainability (CHESS), State Key Laboratory of Earth Surface Processes and Resource Ecology (ESPRE), Beijing Normal University, Beijing 100875, China; 5 School of Natural Resources, Faculty of Geographical Science, Beijing Normal University, Beijing 100875, China

**Keywords:** InVEST model, geographic detector, soil erosion, spatiotemporal variation, Qinghai Lake Basin

## Abstract

The present study aims to quantitatively assess soil erosion intensity (SEI) and amounts in the Qinghai Lake Basin (QLB) over the 1990–2020 period using the Integrated Valuation Ecosystem Services and Tradeoffs (InVEST) model based on multi-source data. In addition, the changing trends and driving factors of soil erosion (SE) in the study area were systematically analyzed. The result showed: (1) An increasing-decreasing trend in the total soil erosion amount (SEA) in the QLB over the 1990–2020 period, with an average SEI of 579.52 t/km^2^. In addition, very low and low erosion classes covered 94.49% of the total surface area, while areas with high SEI were mainly distributed in alpine areas with low vegetation coverage (VC). (2) The highest average SEI was observed in bare land, while grassland and unused land were the main land use (LU) types where SE mainly occurred, with the ratio of the two being 95.78%. (3) The average value of SEI was positively correlated with altitude values below 4800 m. In addition, areas with altitude ranges of 4000–4400 m, 3600–4000 m, and 4400–4800 m were the main areas where SE occurred, with an average total soil erosion ratio (SER) value of 88.73%. (4) The average SEI was directly proportional to the slope degrees. SE occurred mainly in the areas with slope degree ranges of 15–25°, 25–35°, 8–15°, and >35°, accounting for 93.16% of the average total SER value. (5) The q value of the two-factor interaction was greater than that of the single-factor interaction. In addition, the areas with a high SE risk were mainly those with 1220–2510 m rainfall, <0.104 VC, the land use/land cover (LULC) type bare land, the altitude range 4400–4800 m, and a slope of >35°. The interaction between rainfall, VC, LULC, elevation, and slope had a significant impact on the spatial distribution of SEI.

## 1. Introduction

Ecosystems are the basis for the survival and development of human society and essential natural resources. Ecosystems and ecological processes can provide humans with ecological goods and services, ensuring the continuous development of the human living environment [1,2]. However, environmental problems can considerably affect ecological balance and environmental sustainability. Among them, SE is a global environmental issue causing land desertification and soil fertility loss to some extent, requiring effective control measures for SE [3,4,5,6]. Although several SE models have been developed, the Universal Soil Loss Equation (USLE) [5,6] and the Revised Universal Soil Loss Equation (RUSLE) [7,8] have been the most widely applied models in predicting soil losses due to their simple structure, good compatibility with the GIS environment, and ability to accurately reflect regional SE conditions. However, the USLE and RUSLE models can result in erroneous calculations due to the ignorance of the ability of the block to intercept upstream sediments [9]. In contrast, the InVEST model takes into account the block’s ability to intercept upstream sediments, providing SE assessment results with higher accuracy, better visualization, and superior convenience in the calculation of ecosystem services and functions [10].

In recent years, several researchers in China and worldwide have applied the InVEST model to assess soil in numerous study areas. Zhou [11] used the InVEST model to investigate soil conservation in different forest ecosystems in Beijing’s mountainous areas, highlighting the effectiveness of the InVEST model in SE prediction in Beijing’s mountainous areas. Li [12] applied the InVEST model to estimate the amount of soil loss (SL) due to SE in the entire Qinling Mountains area, as well as at the basin and county scales, indicating an estimated SL amount of 1.52 × 10^8^ t, thereby suggesting moderate SE. He [13] used the InVEST model to estimate the SEI in the Qihe River Basin in the Taihang Mountains, China, in 2015 and reported an average SEI of 32.45 t/(hm^2^·a), which was 14.40% different from the measured data, suggesting good estimation results. Zhai [14] used the RUSLE, InVEST, and Unit Stream Power-Based Erosion Deposition (USPED) models to estimate the amount of SL caused by SE in the Loess Plateau over several years, demonstrating the closer estimation results of the InVEST model to the actual sediment yield compared to those of the USLE and USPED models. Han [15] used the InVEST model to estimate the total amount of SE in the low-mountain and hilly areas of Qianxi County in four years, showing an increasing temporal trend in the total amount of SE in the study area. Zhang [16] used the InVEST model to estimate the amount of SL due to SE in the upper reaches of the Miyun Reservoir over the 2000–2019 period and found a decreasing-increasing trend in the total amount of SL. Aneseyee [17] used the InVEST model and sediment transport ratio (SDR) module to estimate the amount of SL and sediment output in different LU types in the Winike watershed of the Omo-Gibe Basin in Ethiopia and showed that the conversion of the forest, grazing, and shrubland areas into cropland accelerated SE in the basin, increasing the total SL amount by 1.76 × 10^8^ t from 1988 to 2018. Marques [18] used the InVEST model to estimate sediment retention in Portugal from 1990 to 2018 and quantitatively analyzed the impact of LU changes on SE, showing consistent simulated SE results with those of the European Soil Data Centre (ESDAC), thereby suggesting the good accuracy of the InVEST model in estimating regional/national SE potentials. To sum up, scholars at home and abroad have used the InVEST model to quantitatively calculate and analyze soil erosion in ecosystems at different regional scales. The effects of its application are good, its precision is high, and the invention of it has had wide application prospects.

The QLB is located at the intersection of the alpine region of the Qinghai–Tibet Plateau (QTP), the northwestern arid region, and the eastern monsoon region in China [19]. The QLB is sensitive to global change and is in an area with a typically fragile ecosystem [20]. Indeed, grasslands in the QLB have begun to degrade in recent years due to global warming and irrational LU by humans. The area ratio of moderate and high SE types in the basin is 83.08%, exhibiting a continuous intensification of SE [21]. However, although some scholars have assessed SE in the QLB, most of them [2,22,23] have used short-term remote sensing data, while only a few scholars have used long-term multi-temporal remote sensing data to estimate the total amount and dynamic changes of SE in the QLB. In this context, the present study aims to estimate the total amount of SE and assess the spatiotemporal dynamic characteristics of SE in the QLB from 1990 to 2020 using the SDR module of the InVEST model. In addition, the geographical detector was used in this study to assess the main factors influencing SE in the study area.

## 2. Materials and Methods

### 2.1. Study Area

**Figure 1:**
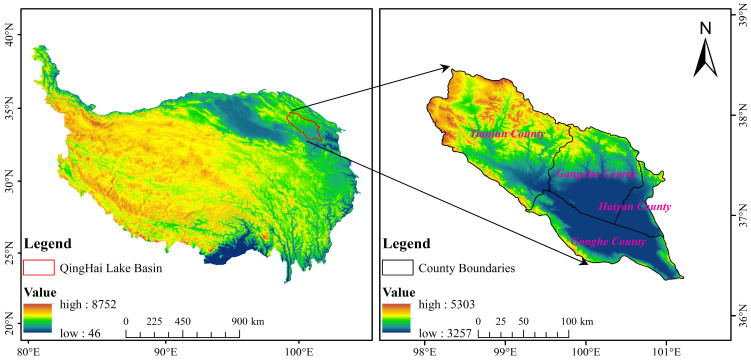
Study area.

The QLB is located in the northeastern part of Qinghai Province on the northeastern edge of the QTP between latitudes and longitudes of 36°15′ N–38°20′ N and 97°50′ E–101°20′ E, respectively. The QLB is the largest inland saltwater lake in China, and it is also a natural barrier preventing the western part from desertification eastward. The QLB is the most important water source and water vapor circulation channel in western China [24]. The QLB covers a total area of 29,669.5 km^2^, exhibiting high and low topography in the northwestern and southeastern parts, with an altitude range of 3257–5303 m. The QLB is characterized by a continental climate, with an average annual temperature range of −4.6–4.0 °C and a large temperature difference between day and night temperatures. Precipitation events occur mainly in the June–August period, with an average annual precipitation range of 291.0–579.0 mm and high solar radiation. The main rivers in the basin are the Buha River, Shaliu River, Hargai River, Heima River, and Quanji River. The vegetation in the QLB consists mainly of grasslands, including temperate and alpine grasslands, dominated by *Achnatherum splendens*, *Stipa purpurea*, and *Kobresia splendens*, whereas the soil types in the QLB are mainly alpine meadow soil, alpine grassland soil, alpine cold desert soil, swamp soil, and aeolian sand soil [2]. The image map of the study area is shown in Figure 1.

### 2.2. Data Sources

**Figure 2:**
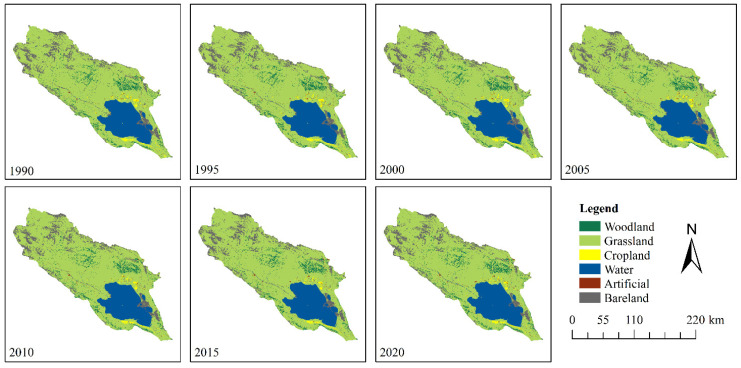
Map of LULC in the QLB.

The data used in this paper and their purpose are listed in Table 1. The LULC results are shown in Figure 2. It should be noted that SoilGrids were fitted in the WoSIS database at a spatial resolution of 250 m based on 230,000 examples of soil profile observation data, with six standard soil layers, and a series of environmental covariates using machine learning algorithms. The new version of SoilGrids is updated and supplemented with soil profile observation data, making it the most comprehensive digital soil mapping system worldwide [25]. Indeed, several researchers have used SoilGrids-derived soil data to investigate SE in several study areas. Mammadli [26] used SoilGrids-derived soil data to estimate the K factor in the South Caucasus, whereas Sourn [27] used SoilGrids to estimate SE in Battambang Province, Cambodia. However, SoilGrids-derived soil data have been rarely used in China.

The range of soil attribute values can be obtained by the weighted average of the values of the depth interval by definite integrals according to the following formula:

(1) $$\frac{1}{b-a}\int_{a}^{b}f(x)dx\approx \frac{1}{b-a}\frac{1}{2}\sum_{k}^{N}\left(x_{k+1-}x_{k}\right)(f(x_{k})+f(x_{k+1}))$$

where *N* denotes the number of soil layers; *k* denotes the serial number of the current soil layer depth; *f*(*x_k_*) denotes the value of the target variable (soil parameter) at *x_k_* depth; *f*(*x_k_*_+1_) denotes the value of the target variable (soil parameter) at *x_k_*_+1_ depth.

### 2.3. Methods

#### 2.3.1. InVEST Model

In this study, we used the SDR module of the InVEST model to calculate the total amount of SL caused by SE in the QLB. The equation for calculating the amount of SE loss by the SDR module is based on that of the *USLE* model, which is expressed as follows:

(2) ULSE=R×K×LS×C×P

where *R* denotes the rainfall erosion factor (MJ·mm/hm^2^); *K* denotes the soil erodibility factor (t·hm^2^·h/MJ·hm^2^·mm); *LS* denotes the slope length and slope factor; *C* represents VC and management factor; *P* denotes soil and water conservation factor.

The SEI in the study area was first classified into six classes based on the SE Classification and Grading Standard [34] and on the actual conditions of the study area, namely of very low erosion (<500 t/km^2^·a), low erosion (500–2500 t/km^2^·a), moderate erosion (2500~5000 t/km^2^·a), high erosion (5000~8000 t/km^2^·a), extreme erosion (8000~15,000 t/km^2^·a), and severe erosion (>15,000 t/km^2^·a). Afterward, the six SEI grades were imported into ArcGIS 10.2 to obtain the SEI map of the QLB.

#### 2.3.2. Input Parameters for Estimating SE

(1) Rainfall erosion factor (*R*)

**Figure 3:**
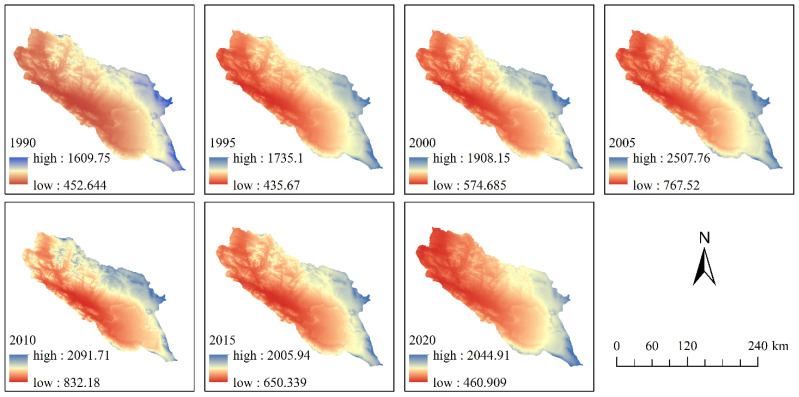
Distribution of rainfall erosivity in the QLB.

The *R* factor reflects the potential ability of rainfall to cause SL. In this study, the 1 km monthly precipitation data (1901–2020) [28,29,30,31] obtained from the National Qinghai–Tibet Plateau Scientific Data Center of China were converted into annual rainfall data after processing (Figure 3). These annual precipitation data were used to calculate the rainfall erosivity according to the following formula [35]:

(3) $$R=\alpha \times P^{\beta }$$

where *R* denotes the average annual rainfall erosivity; *P* denotes the annual rainfall amount (mm); *α* and *β* are formula coefficients, with values of 0.0534 and 1.6548, respectively.

(2) Soil erodibility factor (*K*)

**Figure 4:**
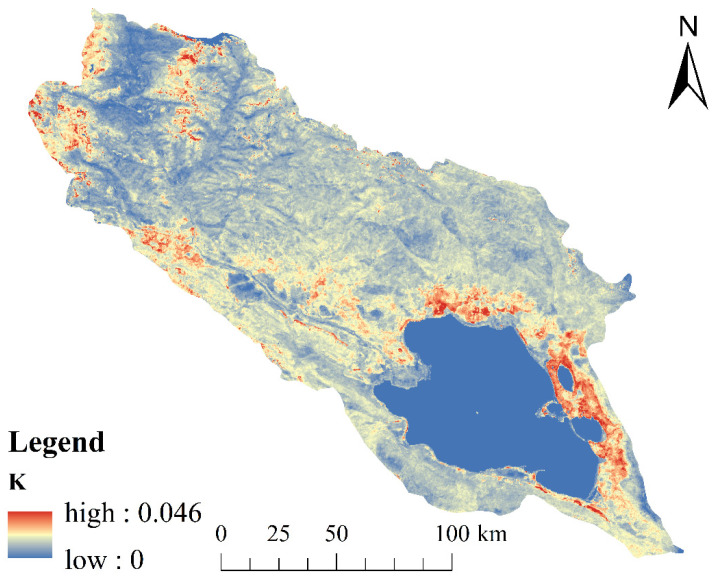
Distribution map of K values in the QLB.

*K* refers to the degree of soil erodibility induced by precipitation, wind, and other external forces. In this study, we used the Environmental Policy Integrated Climate (EPIC) model proposed by Williams et al. [36] to calculate the *K* values of the basin according to the following formulas:

(4) $$SNI=1-\frac{SAN}{100}$$

(5) $$\begin{array}{l} K=0.1317(0.2+0.3\exp(-0.0256SAN(1-SIL/100)))(\frac{SIL}{CLA+SIL}{)}^{0.3}\\ \;(1-\frac{0.25C}{C+\exp(3.72-0.25C)})(1-\frac{0.7SNI}{SNI+\exp(-5.51+22.9SNI)}) \end{array}$$

where *SAN*, *SIL*, *CLA*, and *C* denote the sand, silt, clay, and organic carbon contents (%) in the soil, respectively; 0.1317 represents the conversion factor of *K* values from US units to international units. The calculated *K* (Figure 4) value ranges from 0.024 to 0.046, which is consistent with the *K* range estimated by Liang [37] in Qinghai Province (*K* = 0.015–0.068).

(3) Slope length and slope factor (*LS*)

*LS* reflects the effect of topography on SE. This parameter can be determined using DEM data.

(4) VC and management factors (*C*)

*C* represents the SER under VC to that under bare lands, ranging from 0 to 1. The higher the *C* value, the more serious the SE and the lower the soil holding capacity [38].

Since there is a good correlation between VC and the *C* factor, we referred to the research results of Cai [39] to calculate the *C* values of the basin according to the following formula:

(6) $$C=\left\{\begin{array}{l} 1\\ 0.6508-0.3436\mathrm{lg}(f)\\ 0 \end{array}\right.\begin{array}{l} f=0\\ 0<f\leq 78.3\%\\ f>78.3\% \end{array}$$

where *C* denotes VC and the management factor, and *f* denotes VC (%). *f* can be calculated using the normalized difference vegetation index (*NDVI*) based on Equation (9).

(7) $$f=\frac{NDVI-NDVI_{soil}}{NDVI_{veg}-NDVI_{soil}}$$

where $NDVI_{soil}$ denotes the NDVI value of the bare soil pixel, with a cumulative frequency of 2%, and $NDVI_{veg}$ represents the NDVI value of the vegetation pixel, with a cumulative frequency of 98%.

Similarly to water bodies, the *C* values of building lands were assumed to be 0 since these areas are not affected by SE.

(5) Soil and water conservation measure factor (*P*)

*P* refers to the ratio of the SL amount after implementing SE control measures to that under surface vegetation conditions. Indeed, *P* is an important factor in reducing SE [39]. The *p* values range from 0 to 1, indicating negligible and extreme SE [38], respectively. In this study, the *p* values were determined based on the results reported by Chen [40] and Lin [41] (Table 2).

#### 2.3.3. Geodetector Analysis

Spatial differentiation is a basic characteristic of physical geography. Indeed, the spatial distribution of geographical elements is often uneven, exhibiting spatial heterogeneity [42]. Geographical detectors can be used to detect spatial differentiation, reveal spatial heterogeneity between elements, and quantitatively assess factors influencing geographical phenomena [43]. Geographical detectors consist of risk, ecological, factor, and interaction detectors. The factor detector is mainly used to detect the spatial differentiation of the dependent variable, Y, and assess the degree of influence of factor X on the spatial distribution of Y. It is usually measured by the q-value [44] according to the following equation:

(8) $$q=1-\frac{\sum_{h=1}^{L}N_{h}\sigma_{h}^{2}}{N\sigma^{2}}=1-\frac{SSW}{SST}$$

(9) $$SSW=\sum_{h=1}^{L}N_{h}\sigma_{h}^{2},SST=N\sigma^{2}$$

where h=1; *L* indicates the number of Y or X classes; $N_{h}$ and N denote the number of units in the h layer and the entire watershed, respectively; $\sigma_{h}^{2}$ and $\sigma^{2}$ denote the relative (*h*) and total variances of the layer and watershed, respectively. The *q* value ranges from 0 to 1. The higher and lower the *q* value, the greater and lesser the influences of the factor, respectively.

The interaction detector can identify the interaction between different factors and evaluate the combined effect of independent factors on the explanatory power of the dependent variable or on the independence of the factors. The interaction detector can measure the impacts of factors on the dependent variables by comparing the *q*-values of single and double interaction factors. Risk detectors have been widely used to determine whether there are significant differences in the mean values between the attributes of two sub-regions, whereas the ecological detector is used to determine whether there are significant differences in the influence degrees on the spatial distribution between two factors of the dependent variable. SE is affected by many environmental factors, including climate, vegetation, and topography. Previous studies have assessed SE intensities using geographical detectors based on five factors, namely rainfall, VC, LULC, altitude, and slope [44]. In this study, the multi-year rainfall and VC were classified using the GD package in *R* according to the data discretization method proposed by Wang [45,46]. The slope data were classified into 6 classes according to the surface erosion (sheet erosion) classification standard [34], namely <5°, 5~8°, 8~15°, 15~25°, 25~35°, and >35°. In addition, the altitude values were divided into 5 classes, namely <3600 m, 3600–4400 m, 4000–4400 m, 4400–4800 m, and >4800 m, whereas LU types were divided into woodland, grassland, cropland, water, artificial, and bare land by LULC categories. The QLB was divided into 1 km × 1 km grids, while the SEI, rainfall, VC, LU type, altitude, and slope data of each grid point were used as the operational data of the geographical detector.

## 3. Results

### 3.1. Spatiotemporal Variation Characteristics of SE in the QLB

#### 3.1.1. Temporal Variation Characteristics of SE in the QLB

The SEA in the QLB in 1990, 1995, 2000, 2005, 2010, 2015, and 2020 were 1.38 × 10^8^, 1.88 × 10^8^, 1.95 × 10^8^, 2.29 × 10^8^, 2.25 × 10^8^, 2.02 × 10^8^, and 1.60 × 10^8^ t, respectively. The lowest and highest SEAs were observed in 1990 and 2005, respectively, showing an overall temporally increasing-decreasing trend. In addition, over the 1990–2005 and 2005–2020 periods, the SEA in the QLB increased and decreased by 0.91 × 10^8^ and 0.69 × 10^8^ t, respectively, with an average spatial SEI of 579.52 t/km^2^.

**Figure 5:**
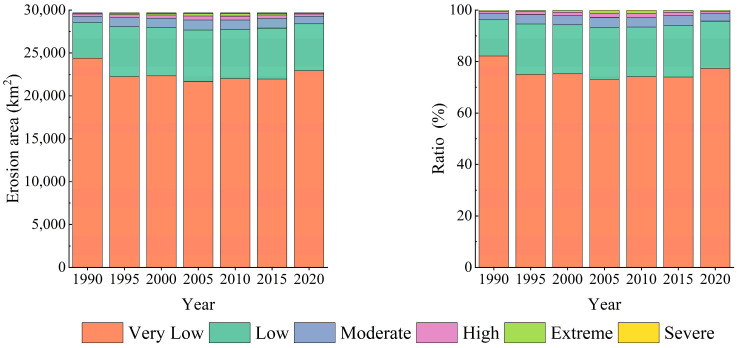
The total amount of SE and its area ratio in the QLB from 1990 to 2020.

The SEA and its area ratios in the QLB basin were determined using ArcGIS and reported in Table 3 and Figure 5. According to the obtained results, very low and low SE areas were the dominant SE classes in the basin, with average annual area ratios of 75.86 and 18.63%, respectively, while the other SEI classes covered relatively small areas. The lowest very low-class ratio in the basin was observed in 2005 (73.13%), showing a decreasing-increasing trend. The highest area ratio of low erosion (20.08%) was observed in 2005, showing a decreasing-increasing trend. In addition, the highest area ratios of moderate and high classes were observed in 2005, showing decreasing-increasing trends, while the highest area ratios of extreme and severe classes were observed in 2010, indicating decreasing-increasing trends.

#### 3.1.2. Spatial Variation Characteristics of SE in the QLB

**Figure 6:**
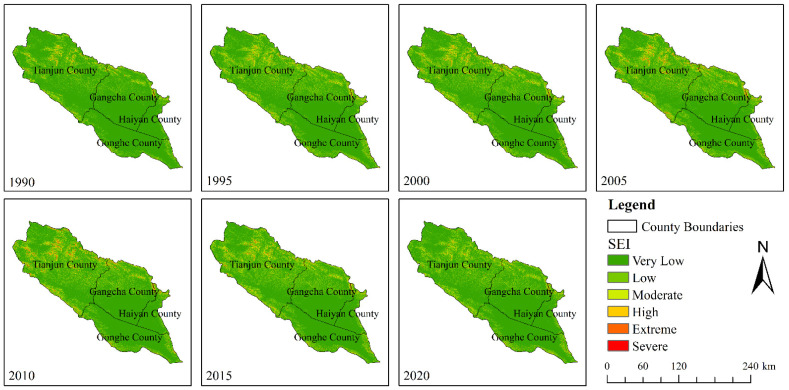
Grading map of SEI in the QLB from 1990 to 2020.

**Figure 7:**
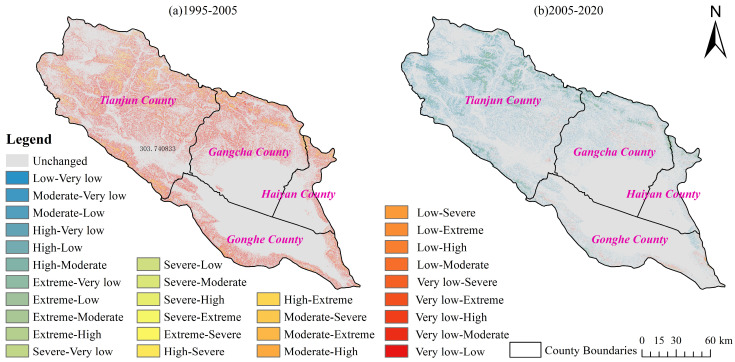
Spatial distribution map of SE grade transfer in the QLB.

The map of SEI classes in the QLB over the 1990–2020 period is shown in Figure 6. It can be seen from Figure 6 that areas with high SEI in Tianjun County were mainly distributed in the central, northern, eastern, and southern parts of the county. In addition, areas with high SEI in Gangcha County were mainly distributed in the northern, northeastern, and northwestern of the county, while those in Haiyan County were mainly distributed in the northern and southeastern parts of the county. Areas with high SEI in Gonghe County were mainly distributed in the southern and eastern regions. The spatial distribution map of SE classes in the QLB is shown in Figure 7. According to Figure 7a and Table 4, it can be seen that from 1990 to 2005, 9.08% of the very low erosion class area in the basin was converted into low erosion areas in several parts of the basin except for flat valleys. Amounts of 0.02, 2.99, 0.03, and 0.01% of the low SE class areas were converted into very low, moderate erosion, high, and extreme SE class areas, respectively. In addition, 0.18% of the extreme SE class area was converted into a severe erosion class area, while no changes in the severe erosion class area were observed. The percentage of the changes from high erosion to low SER and from low erosion intensity to high SER were 0.03 and 14.33%, respectively. Moreover, the total SL amount increased by 0.91 × 10^8^ t in the QLB from 1990 to 2005.

According to Figure 7b and Table 5, 0.21% of the very low SE class area in the basin was converted into low SE class areas over the 2005–2020 period, while 4.39 and 0.05% of the low SE class areas were converted into very low and moderate SE class areas, respectively. In addition, 2.53 and 0.02% of the moderate SE class area was converted into very low and intense SE class areas, respectively. Amounts of 1.32 and 0.01% of the intense SE class areas were converted into moderate and extreme SE class areas, respectively, 0.17 and 0.59% of the extreme SE class areas were converted into moderate and intense SE class areas, respectively, and 0.19% of the severe SE class area was converted into an extreme SE class area. On the other hand, the high-to-low SEI and low-to-high SEI ratios were 9.21 and 0.29%, respectively.

The obtained results demonstrated the conversion of the SEI classes from high to low soil intensity classes, as well as a decrease in the SL amount in the QLB over the 2005–2020 period. These findings might be due to the climatic warming and humidification in the basin and the positive effects of the protection measures in the basin implemented by local governments. The Qinghai Provincial Government formulated the “Regulations on the Protection of the Ecological Environment of the QLB” and “the Qinghai Lake Basin Ecological Environment Protection and Comprehensive Management Policy“ in 2003 and 2008, respectively, which included strengthening the protection and construction of water conservation forests and windbreak and sand-fixation forests in the Qinghai Lake Basin, prohibiting the reclamation of grasslands, returning farmland to grass (forests), and other environmental protection measures.

### 3.2. Impacts of LU Types on SE in the QLB

**Figure 8:**
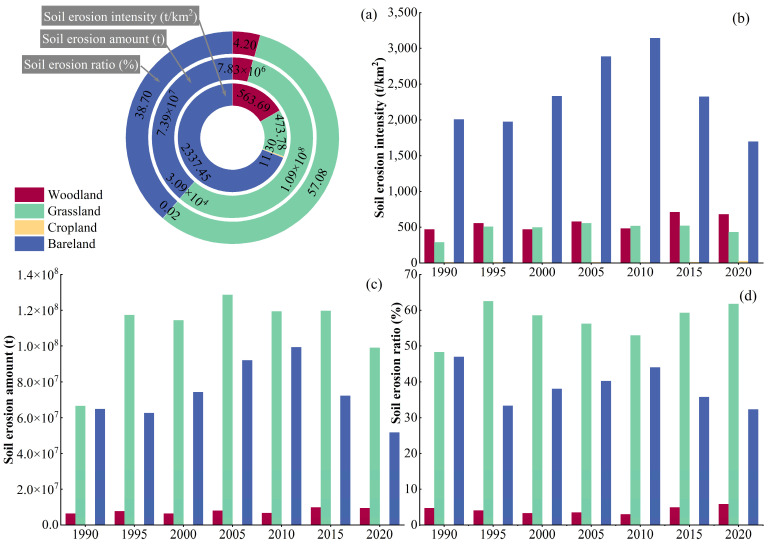
SE characteristics of LU types in the QLB. The average value of SEI for many years (**a**); the SEI from 1990 to 2020 (**b**); the SEA from 1990 to 2020 (**c**); the SER from 1990 to 2020 (**d**).

Human activities make up the main factor affecting LU types, thereby greatly influencing SE rates [10]. To better understand the effects of LU types on SE in the QLB, we superimposed the 1990–2020 SEI map of the study area with the LU type map of the same period. The SEI, total SEA, and total SER of different LU types in the basin were determined using zonal statistics in ArcGIS and reported in Table 6 and Figure 8. The LU types in the watershed were ranked according to the average value of the ratio area in the following order: grassland (69.95%) > water area (15.01%) > bare land (9.58%) > woodland (4.22%) > cropland (0.85%) > construction land (0.40%). Since the *p* factor values of the construction land and water body areas were assumed to be 0, the predicted SEA was 0 t/km^2^. According to the average SE intensities and amounts, the LU types in the basin followed the order bare land > woodland > grassland > cropland and grassland > bare land > woodland > cropland, respective to the SE intensities. The obtained results showed increasing trends in the SE intensities and amounts in woodland and grassland over the 1990–2020 period, while those in cropland and bare land showed decreasing-increasing and decreasing-increasing-decreasing trends, respectively. On the other hand, the order of LU types in the basin according to the different average total SERs were as follows: grassland (57.08%) > bare land (38.70%) > woodland (4.21%) > cropland (0.02%), indicating that grassland and bare land were the main LU types where SE occurred, with a total proportion of 95.78%. In addition, the proportion of SEA in woodland and grassland followed decreasing trends in the 1990–2010 period and increasing trends in the 2010–2020 period. However, due to the small cropland area, no obvious changes in the proportion of SEA in this LU type were observed, with a maximum value of 0.04% in 2020. The proportion of SEA in bare land followed decreasing-increasing-decreasing trends.

### 3.3. Vertical Differences Analysis of SE in the QLB

**Figure 9:**
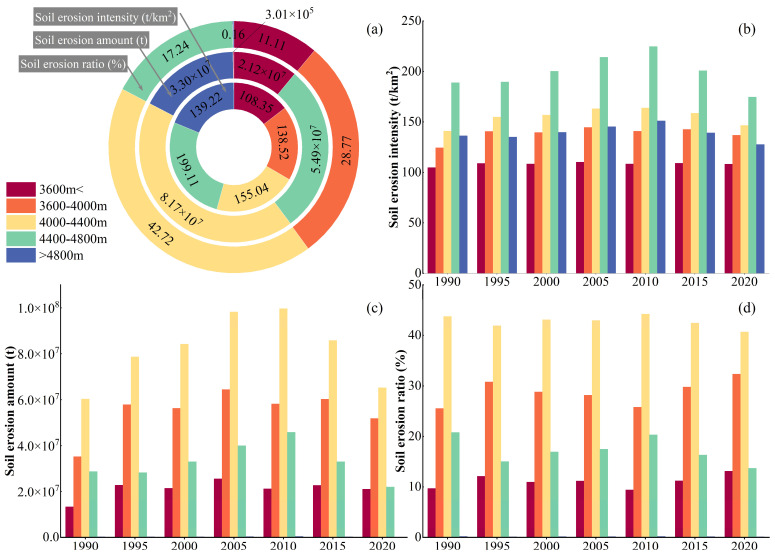
SE characteristics at different altitudes in the QLB. The average value of SEI for many years (**a**); the SEI from 1990 to 2020 (**b**); the SEA from 1990 to 2020 (**c**); the SER from 1990 to 2020 (**d**).

We superimposed five altitude grades with the SE grid map in the study area. The SEI, total SEA, and total SER of different altitudes in the basin were determined using zonal statistics in ArcGIS and are reported in Table 7 and Figure 9.

The altitude classes of the basin were sorted by the mean value of the SE intensities, and were in the following order: 4400–4800 m > 4000–4400 m > above 4800 m > 3600–4000 m > below 3600 m. The average value of SEI in the watershed followed an increasing-decreasing trend with increasing altitude, reaching the highest value at 4400–4800 m.

The results revealed a positive correlation between the mean SEI values and the altitude values above 4800 m. The highest mean SEI values in areas with altitude values below 3600 m and within the 3600–4000 m range were observed in 2005, showing increasing-decreasing trends, whereas the highest mean SEI values at 4000–4400 m, 4400–4800 m, and above 4800 m were observed in 2010, with increasing-decreasing trends.

On the other hand, the order of the altitude ranges of the basin obtained based on the average total SEA was 4000–4400 m > 3600–4000 m > 4400–4800 m > below 3600 m > over 4800 m. The average total SEAs followed increasing-decreasing trends, of which the highest value was observed at 4000–4400 m. The total amount of SE in the areas with altitude ranges below 3600 m and between 3600–4000 m followed increasing-decreasing trends over the 1990–2020 period, reaching the peak values in 2005. The total SEAs in the areas with altitude ranges of 4000–4400 m, 4400–4800 m, and above 4800 m followed increasing-decreasing trends, reaching peak values in 2010. The order of altitude ranges of the basin obtained based on the average total SERs was 4000–4400 m (42.72%) > 3600–4000 m (28.77%) > 4400–4800 m (17.24%) > less than or equal to 3600 m (11.11%) > greater than or equal to 4800 m (0.16%). The areas with the altitude ranges of 4000–4400 m, 3600–4000 m, and 4400–4800 m were the main susceptible areas to SE, with a total average SER of 88.73%. From 1990 to 2020, the changing trends of the total SER in areas with altitude ranges <3600 m and between 3600–4000 m were similar, showing increasing-decreasing-increasing trends, reaching the lowest and highest values in 2010 and 1995, respectively. In addition, the changing trends of the total SERs in the areas with altitude ranges of 4000–4400 m and 4400–4800 m were similar. The average proportion of total SE in the areas with altitude values above 4800 m was 0.16%, exhibiting slight changes and showing the highest and lowest total SE proportion values in 1990 and 2020, respectively.

### 3.4. Impacts of Slope on SE in the QLB

**Figure 10:**
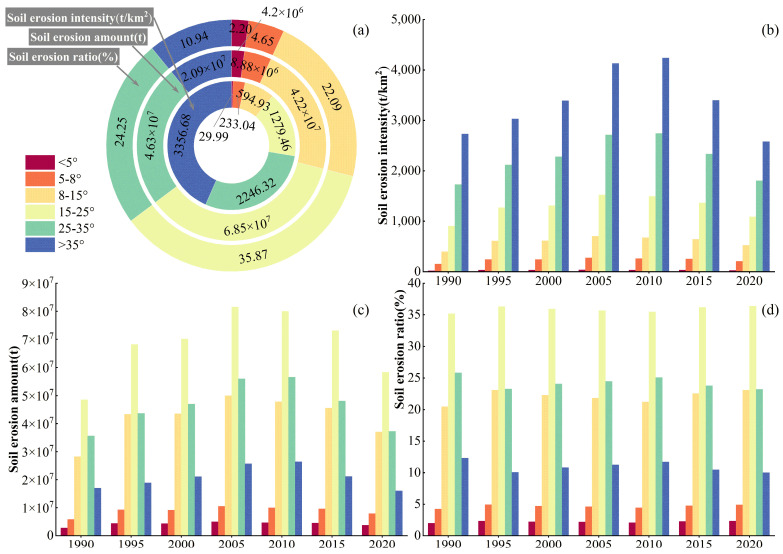
SE characteristics of different slopes in the QLB. The average value of SEI for many years (**a**); the SEI from 1990 to 2020 (**b**); the SEA from 1990 to 2020 (**c**); the SER from 1990 to 2020 (**d**).

The slope is an important factor affecting SE [47], and the six classes of the slope were superimposed with the SE maps, namely those of the SEI, total SEA, and total SER. The proportions of SE in different slope classes in the basin were obtained through zonal statistics in ArcGIS. The obtained statistical results are reported in Table 8 and Figure 10.

It can be seen from Table 8 that the average value of SE intensities is proportional to the slope degrees. The greater the slope, the greater the average value of the SEI. From 1990 to 2020, the mean values of SEIs in areas with the slope degree ranges of <5°, 5–8°, and 8–15° had similar interannual variation trends, showing increasing-decreasing trends and reaching the peak values in 2005, whereas the average value of SEI in areas with slope ranges of 25–35° and >35° exhibited similar changing trends, showing increasing-decreasing trends and reaching peak values in 2010. Although the changing trends of the mean SE intensities at different slopes were similar, the highest SEI values were observed in different years. This result might be due to the serious SE and slow vegetation recovery in the areas of the basin with high slope degrees.

Slope ranges of the basin were sorted by the average values of total SE, and were in the order 15–25° > 25–35° > 8–15° > above 35° > 5–8° > below 5°. From 1990 to 2020, the interannual variation trends of the total SEAs at different slopes were consistent with those of the mean SEI values. The slope ranges of the basin were sorted by the proportions of total SE, and were in the order 15–25° (35.88%) > 25–35° (24.25%) > 8–15° (22.09%) > equal or greater than 35° (10.94%) > 5–8° (4.65%) > equal or less than 5° (2.20%). Therefore, areas with slope ranges of 15–25°, 25–35°, 8–15°, and >35° were the main areas susceptible to SE, accounting for 93.16% of the total SE proportion. In addition, similar interannual trends of the total SE proportions were observed in areas with slope ranges of <5, 5–8, 8–15, and 15–25°over the 1990–2020 period, showing increasing-decreasing-increasing trends. On the other hand, the interannual variation trends of the total SERs at 25–35° and >35° were opposite to those observed at 15–25°, 25–35°, 8–15°, and > 35°, showing decreasing-increasing-decreasing trends.

## 4. Factors Influencing SE in the QLB

To better understand the impacts of environmental factors on the SEI in the QLB, we used geographical detectors to quantitatively assess the main influencing factors. The results showed different degrees of impact of the influencing factors on SEI (Table 9). The main environmental factors influencing SEI in the QLB were in the order slope > LU type > altitude > vegetation cover > rainfall.

The QLB is surrounded by mountains, namely Riyue Mountain, Qinghai Nanshan Mountain, Tianjun Mountain, and Datong Mountain, in the eastern, southern, eastern, and northern parts of the QLB basin [48], respectively. By overlaying the SEI map with each factor map, it was observed that the areas with high average SEIs were mainly distributed in the bare land below the top of the mountain. The average SEI values were positively correlated with the slope degrees. The highest average SEI value was observed in bare land, which is highly consistent with the assumption that bare land is the area with a high average SEI value. Except for cropland with a small area, the average SEI values of woodland and grassland were also high. In addition, the average SEI values in the basin increased with decreasing altitudes below 4800 m, reaching the highest average SEI value at 4400–4800 m. In contrast, an increase in the average value SEI values was observed with increasing altitude values above 4800 m, reducing the explanatory power of the effect of altitude on the SEI in the basin. The LULC, altitude, and slopes were sorted by the mean SEI values, and were in followed the order slope (1290.07 t/km^2^) > LULC (846.56 t/km^2^) > altitude (148.05 t/km^2^). In addition, the results showed a low correlation between VC and SEI in the QLB, which is due to the fact that the areas with high SEIs were basically bare land with low VC values. Indeed, the average VC area covered only 0.07% of bare land. Although rainfall amounts in the QLB decreased from the southeastern to northwestern parts, the areas in the northwestern part of the QLB exhibited high average SEIs, decreasing from the northwestern to southeastern parts of the QLB. These findings demonstrate that the spatial distribution of SE in the QLB is mainly affected by topography and LU types.

The results of the interaction detection are reported in Table 10, indicating higher q values of interaction between influencing factors than those of single factors. In addition, the interaction between factors increased the degree of the effect on SE in the QLB. For example, the slopes had strong effects on SEI, while the q values of the interaction between slope and other factors were all above 0.36. The interaction between slope and LU types were of the highest q-value, 0.4370, which is consistent with the above-mentioned results, indicating that areas with high SEI are located at the intersection of the slope with bare land. The interaction between slope and VC and between slope and rainfall were of q values of 0.3833 and 0.3770, respectively. The results indicated increases in the SEI in areas with low VC and the same slope degree. On the other hand, rainfall exhibited a slight effect on SEI, increasing with increasing SEI, especially in the northeastern part of the basin, which is the area of overlap between rainfall and high erosion intensity. The interaction between slope and altitude was of a a *q* value of 0.3666, indicating an increase in the SEI in high-altitude areas with high slope degrees. The interaction between rainfall and VC had the lowest effects on SEI, with a *q* value of 0.1393, demonstrating that SE occurs in the arid region of northwest China, where rainfall amounts and VC are low.

By comparing the two-factor interaction with the single-factor interaction, the q value of the interaction between slope and rainfall was 7.56 times higher than that of the single-factor *q* value of rainfall, indicating that the interaction effect of rainfall and areas with high slope degrees increases the intensity of SE to a certain extent, whereas the q value of the interaction effect of slope and VC was 5.06 times higher than that of VC, indicating a higher SEI under the same VC and high slope degrees.

The SE risk detection results are reported in Table 11, showing the highest SEI in areas with a rainfall range of 1220–2510 mm, with an average SEI of 218.78 t/km^2^. By overlaying the obtained maps, it can be seen that this area was mainly distributed in the high mountain areas in the eastern part of Gonghe County and the northern part of Haiyan County, which are characterized by high slope degrees and rainfall amounts. The high-risk areas of the VC factor were mainly distributed in areas with values below 0.104, indicating an average SEI of 194.30 t/km^2^. These areas were, indeed, observed in Tianjun County in the northwestern part of the QLB and the high mountainous areas in the northern part of Gangcha County. The areas of high SE risk were mainly distributed in bare land, areas with an altitude range of 4400–4800 m, and areas with slope degrees above 35°, showing average SEI values of 213.52, 200.34 t/km^2^, and 278.28 t/km^2^, respectively.

## 5. Discussion

The geographical detector analysis demonstrated that topographic factors and LU types were the factors influencing SEI in the QLB. This finding might be due to the specific characteristics of the QLB, which is surrounded by mountains, with an altitude range of 3600–5200 m, and steep slopes, promoting SE. The total amount of SE in the study area followed an increasing-decreasing trend over the 1990–2020 period, reaching the peak value in 2005 (2.29 × 10^8^ t). This finding might be due to the combined effects of the climate and human activities.

The QLB is characterized by a typical continental climate, high altitudes, strong winds, sandstorms in spring, low rainfall amounts, and simultaneous rain and heat events. Indeed, about 90% of the rainfall occurs in the May–September period [49]. Moreover, the QLB is characterized by abundant alpine vegetation, resulting in a relatively fragile ecological environment in the QLB to a certain extent. From 1990 to 2020, QLB experienced a warm-dry to warm-humid transition [50]. It should be noted that SEAs have been reduced to some extent as a result of the implementation of ecological and environmental protection policies in the QLB. Indeed, Qinghai Province promulgated the “Regulations on the Protection of the Ecological Environment of the QLB” in August 2003. However, several environmental issues existed in the QLB before the implementation of the watershed protection policy (before August 2003). First, the water level of the QLB continuously declined over the 1961–2003 period, which was mainly controlled by the alternating factors of dry and wet climates [51]. The climate in the QLB was warm and dry in the 1990–2000 period, showing higher evaporation rates than recharge rates of the lake, resulting in a considerable decline in the water level of the lake and, consequently, exposing some lake sediments [52]. Second, the climate in the QLB was warm and dry in the 1990–2000 period, exhibiting severe and slight drought events over four years and one year, respectively, reducing the VC considerably in the basin. Moreover, these severe climate characteristics resulted in the transformation of some vegetation types from medium-wet to medium-dry plants [53]. Precipitation is the main factor influencing vegetation growth in the southeastern part of the basin, while air temperature is the limiting factor for vegetation growth in the northwestern part of the basin [54]. Indeed, precipitation and temperature values exhibited uneven spatial distribution characteristics in the basin, seriously affecting vegetation growth in the basin [54]. Moreover, land reclamation, deforestation, and grazing are also important factors leading to vegetation degradation. Third, vegetation degradation is indirectly caused by an ecological imbalance in the QLB, resulting in several phytosanitary issues in grass, including weeds, rodents, and insects. Due to the partial degradation of pastures, the gradual development of weeds, the over-hunting of rats’ natural enemies, and the overuse of chemical rodent products, the abundance of natural enemies of pikas, such as eagles, mustelids, and foxes, have considerably decreased, resulting in an invasion of a flood of rodents and even high areas of black soil beaches. Fourth, grassland degradation and desertification and the substantial decrease in the water levels of the lake are the main sources of dust in the QLB. Indeed, the warming rate in the QLB is greater than that of the hinterland of the QTP. In addition, the warming rate of the QLB in the cold season is greater than that of the warm season, showing an obvious increase in the temperature in winter [55]. From 1990 to 2000, the climate in the basin was warm and dry. The high warming rates in the cold season caused the breaking up and loosening of rocks and soils in the basin. In addition, the extreme wind events in spring (March–May) resulted in abundant dust events in the basin before rainfall events, thereby increasing the SEAs. Zhang [49] found that the SE rates in the early rainy season (May–June) were higher than those in the middle and late rainy seasons (July–October).

Therefore, in considering the main ecological and environmental problems and the actual situation of SE in the QLB, it is important to conduct further research on SE in the study area, taking into account several factors. In addition, in the context of global climate change, warming, and humidification in the QTP, and of watershed policies, it is important to assess the LU changes, more particularly bare land changes, and their impacts on SE in the QLB. Indeed, it is unclear whether the high SEI in bare land is due to the surrounding mountains or to the less erodible glaciers of bare land. In addition, it is crucial to determine the impacts of soil texture types on SE. On the other hand, rainfall and SEI in the QLB followed opposite spatial distribution trends, suggesting the negligible effect of rainfall on SEI. However, the obtained results showed a high positive correlation between rainfall amounts and SE intensities in the eastern part of the QLB (the eastern part of Gonghe County and the northern mountainous area of Haiyan County). Indeed, this part is characterized by steep slopes, high altitudes, low VC, and high rainfall compared to other parts of the QLB, explaining the higher SEI observed. Therefore, is of great importance to quantify the impacts of rainfall on SE in the context of climatic warming and humidification in the QLB in future studies. In addition, the potential impacts of VC changes in the QTP due to climatic warming and humidification on SE in the watershed need to be further investigated in future studies.

## 6. Conclusions

(1) According to the obtained results, the total SEA in the QLB increased and decreased by 0.91 × 10^8^ and 0.69 × 10^8^ t over the 1990–2005 and 2005–2020 periods, respectively. In addition, 14.33% of the low-level SE area was converted into a high-level SE area, while 0.03% of the high-level SE area was converted into a low-level SE area. On the other hand, 0.29 and 9.21% of the low-level and high-level SE areas were converted into high-level and low-level SE areas, respectively.

(2) From 1990 to 2020, the SE intensities and the total SEAs in the woodlands and grasslands of the QLB followed increasing upward trends, while the proportion of SE followed a decreasing-increasing trend. The SEI and the total SEAs in the cultivated land followed decreasing-increasing trends, while slight variations were observed in the proportion of SE, with the highest value of 0.04% being observed in 2020. The SEI, total SEA, and proportion of SE in bare land followed decreasing-increasing-decreasing trends.

(3) The average SEI and the average total SEAs followed increasing-decreasing trends with increasing altitude. The highest average SEI and SEA values were observed in areas with altitude ranges of 4400–4800 m and 4000–4400 m, respectively.

(4) The results showed increases in the SE intensities with the increasing slope degrees of the basin. In addition, the interannual variation trend of the total SEAs was of a similar interannual variation to that of the average SE intensities at different slopes over the 1990–2020 period.

(5) The results indicated that slope was the main factor influencing SE in the QLB, followed, respectively, by LULC, altitude, vegetation cover, and rainfall. In addition, the results demonstrated that interaction between factors increases their effects on SE in the basin. The calculated q value of the interaction between slope and other factors was higher than 0.36. A risk detection showed that the average SE intensities in areas with 1220–2510 mm rainfall, <0.104VC, the LULC type bare land, 4400–4800 m altitude, and a > 35° slope were 218.78, 194.30, 213.52, 200.34, and 278.28 t/km^2^, respectively.

Based on the above conclusions, this study preliminarily explored the temporal and spatial variation characteristics of soil erosion in the QLB and identified the high-risk areas of soil erosion in the QLB. Various environmental protection policies such as returning farmland to forests and grasslands, returning grazing land to grasslands, prohibiting the private digging of pastures at will, and implementing rotational grazing or rest grazing in pastures in pastoral areas should be continued in the watershed, increasing the vegetation coverage of the soil in the watershed, and enhancing the water and soil conservation capacity of the soil in the watershed. For the areas with high soil erosion risk factors in the watershed, especially in the northwest of the watershed, the strict enforcement of environmental protection policies is required, and excessive grazing and the random excavation of pastures are prohibited. Ecological compensations can be used to subsidize herdsmen who suffer losses caused by policy implementation. For areas with harsh environments that are difficult for manpower to reach, vegetation coverage can be increased by air seeding.

## Figures and Tables

**Table 1:** The data used in this study.

Data	Date Resource	Resolution	Year	Purpose
DEM	Geospatial Data Cloud (https://www.gscloud.cn/ (accessed on 1 June 2022))	30 m	2011	model LS calculation, extraction of elevation, and slope
LULC	United States Geological Survey (https://glovis.usgs.gov (accessed on 1 June 2022))	30 m	1990, 1995, 2000, 2005, 2010, 2015, 2020	providing the LU type data
R [28,29,30,31]	The National Qinghai–Tibet Plateau Scientific Data Center of China (https://data.tpdc.ac.cn (accessed on 1 June 2022))	1000 m	1990, 1995, 2000, 2005, 2010, 2015, 2020	extracting the mean value of the dataset to generate the mean annual rainfall
SoilGrids [32,33]	The International Soil Information Center	250 m	2020	calculating the soil erodibility factor (K)

**Table 2:** Factor values of *P*.

Type	Woodland	Grassland	Cropland	Water	Artificial	Bareland
*P*	1	1	0.4	0	0	1

**Table 3:** Areas and proportions of six types of SE in the QLB from 1990 to 2020.

SEI	Year	SE Area/km^2^	SER/%	SEI	Year	SE Area/km^2^	SER/%
Very Low	1990	24,388.95	82.20	Low	1990	4163.08	14.03
	1995	22,251.17	75.00		1995	5814.87	19.60
	2000	22,324.74	75.24		2000	5650.18	19.04
	2005	21,697.51	73.13		2005	5957.07	20.08
	2010	21,997.65	74.14		2010	5710.37	19.25
	2015	21,959.54	74.01		2015	5945.40	20.04
	2020	22,937.86	77.31		2020	5454.76	18.39
Moderate	1990	729.84	2.46	High	1990	246.97	0.83
	1995	1068.60	3.60		1995	334.88	1.13
	2000	1074.96	3.62		2000	375.68	1.27
	2005	1190.54	4.01		2005	464.87	1.57
	2010	1123.76	3.79		2010	464.21	1.56
	2015	1136.78	3.83		2015	383.22	1.29
	2020	889.98	3.00		2020	251.01	0.85
Extreme	1990	114.61	0.39	Severe	1990	26.05	0.09
	1995	161.07	0.54		1995	38.91	0.13
	2000	194.86	0.66		2000	49.07	0.17
	2005	277.22	0.93		2005	82.28	0.28
	2010	286.22	0.96		2010	87.28	0.29
	2015	195.37	0.66		2015	49.19	0.17
	2020	111.31	0.38		2020	24.57	0.08

**Table 4:** The transfer matrix of SEI in the QLB in 1990–2005 (%).

1990–2005	Very low	Low	Moderate	High	Extreme	Severe
Very low	73.11	9.08	0.00	-	-	-
Low	0.02	10.99	2.99	0.03	0.01	0.00
Moderate	0.00	0.01	1.02	1.31	0.13	0.00
High	0.00	0.00	0.00	0.23	0.59	0.01
Extreme	0.00	0.00	0.00	0.00	0.21	0.18
Severe	-	-	0.00	0.00	0.00	0.09

**Table 5:** Transfer matrix of SEI in QLB in 2005–2020 (%).

2005–2020	Very low	Low	Moderate	High	Extreme	Severe
Very low	72.92	0.21	0.00	0.00	0.00	-
Low	4.39	15.63	0.05	0.00	0.00	-
Moderate	0.00	2.53	1.46	0.02	0.00	0.00
High	0.00	0.00	1.32	0.23	0.01	0.00
Extreme	0.00	0.00	0.17	0.59	0.17	0.00
Severe	0.00	-	0.00	0.00	0.19	0.08

**Table 6:** SEI, SEA, and SER of LU types.

Index	LULC	Multi-Year Average	1990	1995	2000	2005	2010	2015	2020
SEI (t/km^2^)	Woodland	563.69	469.86	554.08	469.18	579.34	482.32	711.73	679.31
	Grassland	473.78	289.23	508.43	495.87	556.24	515.74	519.11	431.87
	Cropland	11.30	8.98	8.30	6.96	8.11	5.70	14.71	26.34
	Bare land	2337.45	2005.74	1975.00	2332.13	2887.17	3143.45	2322.53	1696.16
SEA (t)	Woodland	7.83 × 10^6^	6.51 × 10^6^	7.68 × 10^6^	6.51 × 10^6^	8.05 × 10^6^	6.72 × 10^6^	9.91 × 10^6^	9.46 × 10^6^
	Grassland	1.09 × 10^8^	6.66 × 10^7^	1.17 × 10^8^	1.14 × 10^8^	1.29 × 10^8^	1.19 × 10^8^	1.20 × 10^8^	9.90 × 10^7^
	Cropland	3.09 × 10^4^	2.79 × 10^4^	2.58 × 10^4^	2.22 × 10^4^	2.20 × 10^4^	1.42 × 10^4^	3.68 × 10^4^	6.77 × 10^4^
	Bare land	7.39 × 10^7^	6.48 × 10^7^	6.26 × 10^7^	7.44 × 10^7^	9.20 × 10^7^	9.93 × 10^7^	7.24 × 10^7^	5.18 × 10^7^
SER (%)	Woodland	4.20	4.72	4.10	3.33	3.52	2.98	4.91	5.90
	Grassland	57.08	48.28	62.53	58.58	56.22	52.93	59.26	61.74
	Cropland	0.02	0.02	0.01	0.01	0.01	0.01	0.02	0.04
	Bare land	38.70	46.98	33.37	38.08	40.25	44.08	35.81	32.31

**Table 7:** SEI, SEA, and SER at different altitudes.

Index	Dem (m)	Multi-Year Average	1990	1995	2000	2005	2010	2015	2020
SEI (t/km^2^)	3600 m<	147.76	93.43	158.85	149.70	178.67	148.23	158.44	147.03
	3600–4000 m	633.11	406.79	666.98	649.49	743.87	671.16	695.18	598.29
	4000–4400 m	992.33	733.09	954.45	1022.35	1192.40	1210.80	1041.18	792.02
	4400–4800 m	1940.66	1686.74	1661.05	1945.05	2355.97	2696.12	1945.13	1294.60
	>4800 m	708.26	633.25	606.80	709.40	852.43	1016.95	699.91	439.07
SEA (t)	3600 m<	2.12 × 10^7^	1.34 × 10^7^	2.27 × 10^7^	2.14 × 10^7^	2.56 × 10^7^	2.12 × 10^7^	2.27 × 10^7^	2.11 × 10^7^
	3600–4000 m	5.49 × 10^7^	3.53 × 107	5.78 × 10^7^	5.63 × 10^7^	6.45 × 10^7^	5.82 × 10^7^	6.03 × 10^7^	5.19 × 10^7^
	4000–4400 m	8.17 × 10^7^	6.04 × 10^7^	7.86 × 10^7^	8.42 × 10^7^	9.82 × 10^7^	9.97 × 10^7^	8.57 × 10^7^	6.52 × 10^7^
	4400–4800 m	3.30 × 10^7^	2.87 × 10^7^	2.82 × 10^7^	3.31 × 10^7^	4.00 × 10^7^	4.58 × 10^7^	3.31 × 10^7^	2.20 × 10^7^
	>4800 m	3.01 × 10^5^	2.69 × 10^5^	2.58 × 10^5^	3.02 × 10^5^	3.62 × 10^5^	4.32 × 10^5^	2.98 × 10^5^	1.87 × 10^5^
SER (%)	3600 m<	11.11	9.70	12.12	10.98	11.19	9.42	11.23	13.13
	3600–4000 m	28.77	25.56	30.81	28.83	28.20	25.82	29.83	32.35
	4000–4400 m	42.72	43.76	41.89	43.11	42.94	44.24	42.44	40.68
	4400–4800 m	17.24	20.78	15.04	16.93	17.51	20.33	16.36	13.72
	>4800 m	0.16	0.20	0.14	0.15	0.16	0.19	0.15	0.12

**Table 8:** SEI, SEA, and SER of different slopes in the QLB.

Index	Slope	Multi-Year Average	1990	1995	2000	2005	2010	2015	2020
SEI (t/km^2^)	<5°	29.99	19.54	31.28	31.01	35.55	33.43	32.42	26.71
	5–8°	233.04	152.62	242.27	241.03	275.69	261.00	252.01	206.67
	8–15°	594.93	398.04	610.79	613.44	703.77	674.17	642.02	522.30
	15–25°	1279.46	905.75	1272.24	1309.21	1522.57	1492.60	1364.59	1089.28
	25–35°	2246.32	1728.12	2118.87	2279.43	2715.06	2743.61	2332.50	1806.65
	>35°	3356.68	2731.46	3030.00	3390.02	4129.40	4238.33	3398.84	2578.75
SE (t)	<5°	4.20 × 10^6^	2.74 × 10^6^	4.38 × 10^6^	4.3 × 10^6^	4.98 × 10^6^	4.69 × 10^6^	4.54 × 10^6^	3.74 × 10^6^
	5–8°	8.88 × 10^6^	5.82 × 10^6^	9.23 × 10^6^	9.19 × 10^6^	1.05 × 10^7^	9.95 × 10^6^	9.61 × 10^6^	7.88 × 10^6^
	8–15°	4.22 × 10^7^	2.82 × 10^7^	4.33 × 10^7^	4.35 × 10^7^	4.99 × 10^7^	4.78 × 10^7^	4.56 × 10^7^	3.71 × 10^7^
	15–25°	6.85 × 10^7^	4.85 × 10^7^	6.82 × 10^7^	7.01 × 10^7^	8.16 × 10^7^	8.00 × 10^7^	7.31 × 10^7^	5.84 × 10^7^
	25–35°	4.63 × 10^7^	3.56 × 10^7^	4.37 × 10^7^	4.70 × 10^7^	5.60 × 10^7^	5.66 × 10^7^	4.81 × 10^7^	3.72 × 10^7^
	>35°	2.09 × 10^7^	1.70 × 10^7^	1.89 × 10^7^	2.11 × 10^7^	2.57 × 10^7^	2.64 × 10^7^	2.11 × 10^7^	1.60 × 10^7^
SER (%)	<5°	2.20	1.99	2.34	2.23	2.18	2.08	2.25	2.33
	5–8°	4.65	4.22	4.92	4.70	4.60	4.41	4.75	4.91
	8–15°	22.09	20.48	23.10	22.29	21.84	21.23	22.55	23.12
	15–25°	35.87	35.18	36.32	35.92	35.67	35.48	36.18	36.40
	25–35°	24.25	25.82	23.27	24.06	24.47	25.09	23.80	23.23
	>35°	10.94	12.32	10.05	10.80	11.24	11.70	10.47	10.01

**Table 9:** Detection of SE factors in the QLB from 1990 to 2020.

	Variable	q
1	Slope	0.3298
2	LULC	0.1399
3	DEM	0.0995
4	C	0.0756
5	R	0.0499

**Table 10:** The q value of the interaction of each factor on SE in the QLB.

	R	C	LULC	Dem	Slope
R	0.0499	0.1393	0.1924	0.1721	0.3770
C	0.1393	0.0756	0.1459	0.1461	0.3833
LULC	0.1924	0.1459	0.1399	0.2178	0.4370
DEM	0.1721	0.1461	0.2178	0.0995	0.3666
Slope	0.3770	0.3833	0.4370	0.3666	0.3298

**Table 11:** Risk detection value of each factor of SEI in QLB.

Impact Factor	R	C	LULC	Dem	Slope
High-risk areas	1220–2510 mm	<0.104	Bareland	4400–4800	>35°
Mean SEI	218.78	194.30	213.52	200.34	278.28

## Data Availability

Research data can be obtained from the corresponding author through email.
